# Catechin-rich green tea extract increases serum cholesterol levels in normal diet- and high fat diet-fed rats

**DOI:** 10.1186/1753-6561-6-S3-P47

**Published:** 2012-06-01

**Authors:** Toshikazu Suzuki, Ayumi Takagi, Michiyo Takahashi

**Affiliations:** 1 Department of Health and Nutrition, Wayo Women’s University, Ichikawa, Chiba 272-8533, Japan; 2 Graduate School of Human Ecology, Wayo Women’s University, Ichikawa, Chiba 272-8533, Japan

## Background

*In vivo* studies using rodents have shown that green tea extract and catechins isolated from green tea can induce a variety of health effects, including anti-obesity, hypoglycemic and hypolipidemic activities [1]. These beneficial effects of green tea have been observed in experiments using high fat, high cholesterol and high fructose diet-fed animals. In the present study, we examined the effects of catechinrich green tea extract on serum glucose and lipid levels in normal diet-and high fat diet-fed rats.

## Materials and methods

Catechin-rich (30% catechin) green tea extract (GT) was used for *in vivo* studies. Male Sprague-Dawley rats (average weight 232.9 g) were divided into six groups containing six rats each. The first group was fed on a normal diet (ND, 10% calories from fat); the second group on a high fat diet (HFD, 40% calories from fat); third group with ND containing 1% GT(ND + 1% GT); fourth group with HFD + 1% GT; fifth group with ND + 3% GT; and the sixth group with HFD + 3% GT. After four weeks of feeding, rats were euthanized by whole blood collection under anesthesia. Total RNA samples extracted from the liver were used for microarray analysis.

## Results

Body weight was significantly lower in GT-containing diet-fed rats than that in GT-free diet-fed rats regardless of whether they received ND or HFD (Table 1). As expected, GT reduced serum glucose (Glc) and triglycerides (TG) levels in ND and HFD-fed rats but was GT concentration dependent (Table 1). Diets containing 1% GT did not affect the serum levels of total cholesterol (T-Cho) and high-density lipoprotein cholesterol (HDL-Cho) significantly, although there was a trend towards an increase in cholesterol levels. When 3% GT was added to the diet, the serum levels of T-Cho and HDL-Cho increased significantly in ND and HFD-fed rats compared to non-GT fed rats (Table 1). The degree of increase in the levels of these serum factors was higher in ND-fed rats compared with HFD-fed rats. Serum AST and ALT levels suggested that hepatic damage induced by GT feeding had not occurred (Table 1). Preliminary microarray analysis data suggested that mRNA levels of more than half of the genes involved in cholesterol synthesis were increased and the mRNA levels of *Cyp7a1*, which is involved in bile acid synthesis, was decreased in ND + 3% GT-fed rats compared with GT-free ND-fed rats.

**Table 1:** Impact of green tea extract on body weight, liver weight, serum glucose, serum lipids, serum AST, and serum ALT in rats fed an ND or a HFD diet ^1^

Variables	ND	HFD	ND + 1% GT	HFD + 1% GT	ND + 3% GT	HFD + 3% GT
Body weight (g)	370±174	417 ± 11.1*	347 ± 9.8^*,‡^	399 ± 12.6^*,‡^	309 ± 9.1^*,‡^	341 ± 18.1^*,‡^
Liver weight (g)	11.1 ± 1.0	12.9 ± 1.8	9.4 ± 1.1^*,‡^	11.1 ± 1.1	8.2 ± 0.3^*,‡^	9.0 ± 0.6^*,‡^
Glc (mg/dL)	200 ± 29.6	184 ± 29.5	167 ± 40.2	156 ± 37.4*	131 ± 22.5^*,‡^	140 ± 22.9^*,‡^
TG(mg/dL)	52.6 ± 24.5	76.3 ± 12.2	39.8 ± 5.9^‡^	52.3 ± 11.6^‡^	30.2 ± 21.6^*,‡^	38.5 ± 7.2^*,‡^
T-Cho (mg/dL)	57.1 ± 6.1	55.2 ± 5.8	51.0 ± 8.5	55.2 ± 6.9	84.6 ± 7.3^*,‡^	70.3 ± 9.4^*,‡^
HDL-Cho (mg/dL)	36.3 ± 6.0	33.4 ± 5.9	38.3 ± 5.6	38.0 ± 3.9	58.8 ± 11.0^*,‡^	46.7 ± 7.6^*,‡^
AST (u/l)	94.3 ± 32.1	87.5 ± 30.5	88.7 ± 23.8	71.8 ± 25.6	78.7 ± 11.4	69.0 ± 15.6
alt (u/l)	12.0 ± 2.5	14.0 ± 5.5	13.2 ± 2.1	10.5 ± 5.2	17.5 ± 3.1*	15.0 ± 3.8

^1^Data are means ± SD, n=6. *p < 0.05 versus ND. ^‡^*p* < 0.05 versus HFD,

## Conclusions

The results from the current study suggest that GT can increase serum cholesterol levels, especially in ND-fed rats, when it is consumed in excess. This effect may partly occur through changes in liver gene expression induced by GT feeding. Further studies are required to evaluate whether the effects of GT are beneficial or harmful to health.
